# Mechanical and optical properties of conventional restorative glass-ionomer cements - a systematic review

**DOI:** 10.1590/1678-7757-2018-0357

**Published:** 2019-02-21

**Authors:** Rafael MENEZES-SILVA, Renata Nunes CABRAL, Renata Corrêa PASCOTTO, Ana Flávia Sanches BORGES, Carolina Castro MARTINS, Maria Fidela de Lima NAVARRO, Sharanbir K. SIDHU, Soraya Coelho LEAL

**Affiliations:** 1 Faculdade de Odontologia de Bauru, Departamento de Dentística, Endodontia e Materiais Odontológicos, Bauru, SP, Brasil.; 2 Universidade de Brasília, Departamento de Odontologia, Brasília, DF, Brasil.; 3 Universidade Estadual de Maringá, Departamento de Odontologia, Maringá, PR, Brasil.; 4 Faculdade de Odontologia da Universidade Federal de Minas Gerais, Departamento de Odontopediatria e Ortodontia, Belo Horizonte, MG, Brasil.; 5 Queen Mary University of London, Institute of Dentistry, London, United Kingdom.

**Keywords:** Glass-ionomer cement, Mechanical properties, Optical properties, Restoration

## Abstract

**Objectives:**

To perform a systematic review of test methodologies on conventional restorative glass-ionomer cement (GIC) materials for mechanical and optical properties to compare the results between different GICs.

**Material and Methods:**

Screening of titles and abstracts, data extraction, and quality assessments of full-texts were conducted in search for *in vitro* studies on conventional GICs that follow the relevant specifications of ISO standards regarding the following mechanical and optical properties: compressive strength, flexural strength, color, opacity and radiopacity.

**Sources:**

The Latin American and Caribbean Health Sciences (LILACS), Brazilian Bibliography of Dentistry (BBO) databases from Latin-American and Caribbean System on Health Sciences Information (BIREME) and PubMed/Medline (US National Library of Medicine - National Institutes of Health) databases were searched regardless of language. Altogether, 1146 *in vitro* studies were selected. Two reviewers independently selected and assessed the articles according to pre-established inclusion/exclusion criteria. Among all the properties investigated, only one study was classified as being of fair quality that tested compressive strength and was included. It was observed that many authors had not strictly followed ISO recommendations and that, for some properties (diametral tensile strength and microhardness), there are no guidelines provided.

**Conclusions:**

It was not possible to compare the results for the mechanical and optical properties of conventional restorative GICs due to the lack of standardization of studies.

## Introduction

Since the 1950s, when the deleterious effects of mercury on humans became known, a worldwide movement to control and reduce its use in a variety of products, processes, and industries was observed^1^. These actions culminated, with the signing of the Treaty of Minamata by 128 countries in October 2013, with the aim of reducing the atmospheric emissions of the mercury through environmental practices and the best available techniques for new enterprises^2^.

Currently, the two main direct dental materials available in oral health as alternatives to amalgam restorations are resinous materials and polyalkenoate-based materials, among which the most biomimetic material is the glass-ionomer cement^3^.

The main advantages of composite resins are their excellent mechanical properties, good aesthetics and handling, which contribute to a reduced operative time^4^. On the other hand, the two most critical problems associated with such aesthetic restorations are the absence of therapeutic remineralization of the carious dentin and the low durability/integrity of the resin-dentin interface over time^5^.

In contrast, glass-ionomer cements (GICs) have interesting properties such as biocompatibility, bioactivity, fluoride release, excellent coefficient of linear thermal expansion/contraction and modulus of elasticity, as well as being the only restorative material capable of chemically bonding to the tooth structure^6^.

However, the first restorative GICs had insufficient mechanical properties to be indicated as definitive posterior and anterior restorations in permanent teeth^7^ ^,^ ^8^. In order to overcome their poor mechanical properties, various modifications have been added into the cement powder and liquid, such as bioactive apatite, zirconia, zinc, strontium oxide, fibers, stainless steel, silica, nanocrystals, among others^9^ ^-^ ^17^. As a result of these improvements, glass-ionomer cement may now be indicated for posterior and anterior restorations of deciduous and permanent teeth^6^ ^,^ ^18^.

Due to the wide variety of dental products that are constantly being launched in the world market, selecting the ideal restorative material becomes a difficult task for the clinician. Prior to clinical trials, laboratory tests are of fundamental importance in guiding professionals regarding the choice of material for their daily practice, as they test the effects of material composition changes or the evolution of their properties and can predict their clinical performance^19^.

The International Organization for Standardization (ISO) was established in 1947 with the aim of approving international standards in all technical fields, including dentistry. The ISO provides quality assurance for dental materials through regulation and standardization of tests that evaluate materials, ensuring their reproducibility in different centers^20^.

It has been observed that many studies which have previously evaluated the properties of GICs did not follow a standardized protocol, such as environment temperature of specimen storage, duration of storage, size of the specimens and the load applied in the tests. All these factors are associated with variation in the manipulation technique in different centers and the lack of standardization on reporting *in vitro* studies and the powder/liquid ratio of the cements make it impossible to compare and discuss the results in literature^21^ ^-^ ^23^.

Hence, a systematic review of literature is necessary in order to verify the standardization in laboratory tests performed with these materials.

Therefore, the objectives of this review were (1) to systematically analyze the conventional restorative GIC test methodologies for the following properties: compressive strength, flexural strength, color, opacity and radiopacity; and (2) to compare the above-mentioned properties of different GICs.

## Material and methods

### Protocol and registration

The protocol of this systematic review is registered at the Prospective International Registration of Systematic Reviews (PROSPERO) (https://www.crd.york.ac.uk/PROSPERO/) with reference number CRD42017050061. It aims to answer the following PICO question: Is it possible to compare the results of mechanical and optical properties of conventional restorative GICs obtained from laboratory studies?

### Eligibility criteria

#### Inclusion criteria

*In vitro* studies published from 1990 onwards which reported on mechanical and optical properties (compressive strength, flexural strength, color, opacity, radiopacity) of conventional glass-ionomer cements were considered eligible. There was no language restriction.

#### Exclusion criteria

Studies were excluded if: 1) the number of specimens *per* group was less than five; 2) there was incorrect or missing statistical analysis; 3) the use of resin-modified glass-ionomer cement was reported; 4) it did not follow the recommendations of the ISO standards 9917-1 for compressive strength, color, opacity and radiopacity and 9917-2 and 4049 for flexural strength^24^ ^-^ ^26^. Whenever ISO established a range for the tests, the value considered was the mean value. For example, for the compressive strength test, it is established that the mechanical tester should be operated at a cross-head speed of (0.75±0.30) mm/min, the value considered was 0.75 mm/min.

## Databases and search strategies

**Figure 1:**
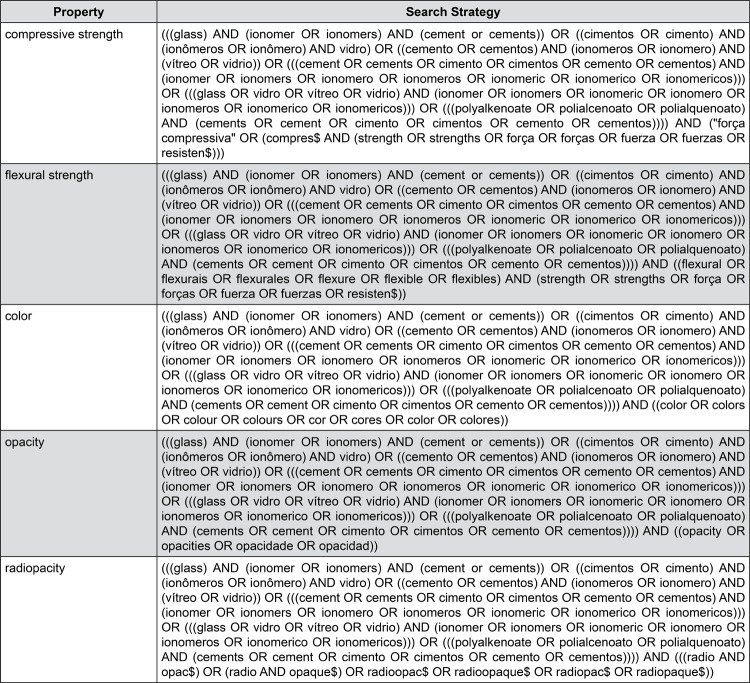
Search Strategy used for LILACS and BBO (Latin-American and Caribbean System on Health Sciences Information)

**Figure 2:**
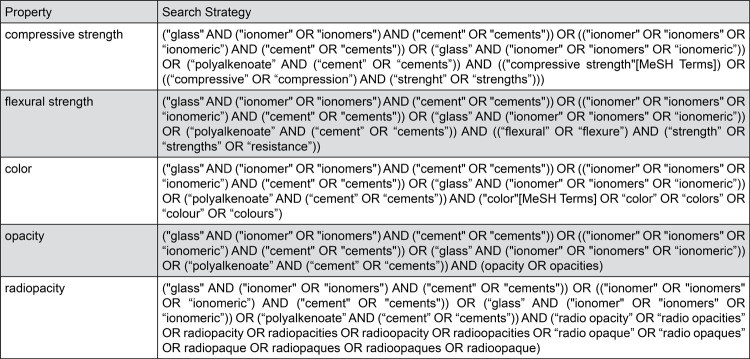
Search strategy used for PubMed/Medline (US National Library of Medicine - National Institutes of Health)

The literature search was conducted on the Latin American and Caribbean Health Sciences (LILACS) and Brazilian Bibliography of Dentistry (BBO) databases from the Latin-American and Caribbean System on Health Sciences Information (BIREME) on February 28^th^, 2018, as well as PubMed/Medline (US National Library of Medicine - National Institutes of Health) on March 1^st^, 2018. On the basis of the properties of conventional glass-ionomers assessed in this review — compressive strength, flexural strength, color, opacity and radiopacity — the search strategies were conducted using controlled vocabulary (MeSH terms/DeCS) and free keywords in English (PubMed, LILACS, BBO), Portuguese and Spanish (LILACS, BBO) as shown in Figures 1 and 2.

## Selection of studies and calibration of investigators

Initially, the abstracts and titles of the studies identified by the search strategy were screened by two independent investigators in order to select the studies that would be fully read. The studies selected were then independently analyzed by the same two investigators in order to check their eligibility according to the inclusion and exclusion criteria. In case of disagreement between the investigators, a consensus was reached through discussion with external consultation.

## Risk of bias and quality of evidence

Data extraction and quality assessment of the included studies were critically evaluated by two independent investigators. For quality assessment, the following variables were analyzed according to the CRIS guidelines^22^ for *in vitro* studies: 1) sample preparation and handling; 2) allocation sequence and randomization process; 3) whether the evaluators were blinded; and 4) statistical analysis. Studies with information about all variables were deemed to be of good quality; if 2- 3 variables were present, they were deemed of fair quality; and lastly, they were classified as being of poor quality when none or just one aspect was covered.

## Results

### Search and included studies

**Figure 3:**
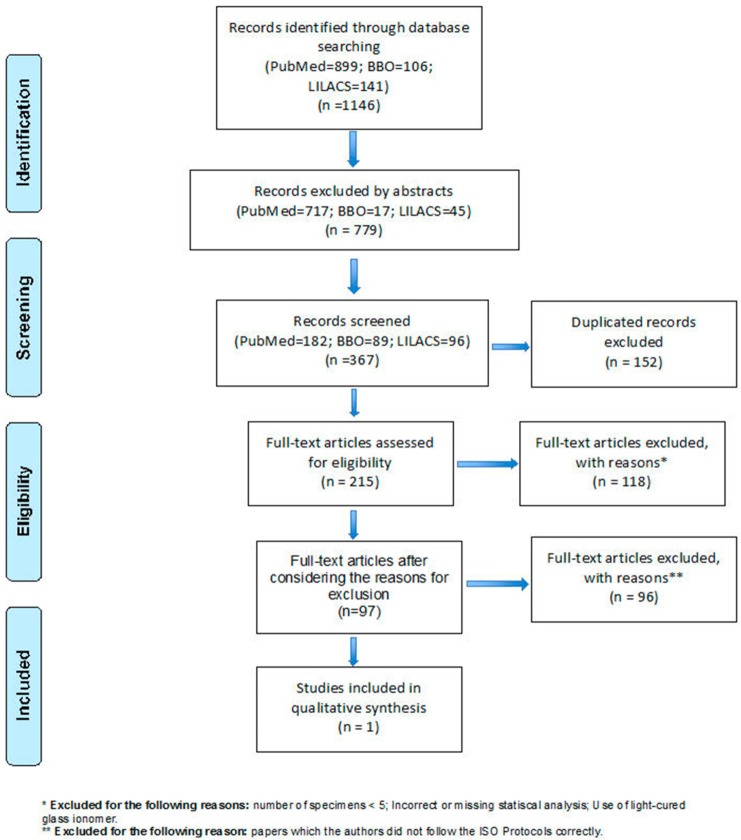
Flowchart showing the inclusion process of the studies that composed in the review

Initially, 1146 studies were found, but only 367 articles were selected after screening the titles and abstracts. From these, 152 duplicated articles were excluded. Eventually, 215 articles were fully read, from which 118 were excluded due to the main exclusion criteria and 97 were excluded by not following the ISO Protocols. The number of articles excluded according to the ISO recommendation are shown in Table 1. Considering the distribution of studies according to the properties tested, it was observed that only one article about compressive strength was included, while none on flexural strength, color, opacity and radiopacity were according to our criteria (Figure 3).

**Table 1:** Number* of studies excluded according to the ISO recommendations by property

ISO recommendations	Mechanical and optical properties				
	Compressive strength	Flexural strength	Color	Opacity	Radiopacity
Specimens´ dimension	27	23	5		5
Equipment test speed	34	23			
Storage time	4		13		
Storage temperature	5				
Focus-film distance					1

*The total number of papers presented in the table is higher than 96 that were excluded as some papers did not follow more than one recommendation

**Figure 4:**
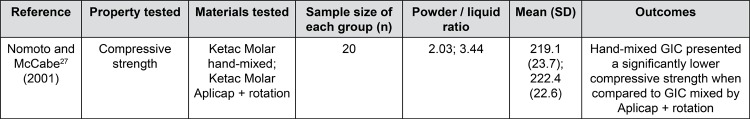
Characteristics of the study included in the review according to the property tested

The characteristics of the study included are shown in Figure 4. Comparisons among studies were thus not possible.

With respect to the quality assessment, the only study included was considered of fair quality, as the authors did not inform whether evaluators were blinded to the type of procedure or material that the sample was subjected to and how the samples were allocated to the different groups studied.

## Discussion

In the field of dental materials, when a new product is finally tested in clinical trials, a series of laboratory tests would have already been carried out in order to verify its microstructure, properties and handling characteristics^28^. However, as no dental material currently available in the market has ideal properties for any dental application^29^ ^,^ ^30^, the industry/researchers are constantly searching for improvements. This requires, among other aspects, comparisons of laboratory test results of products from different generations, which is only possible if such tests follow standardized protocols. Therefore, this systematic review aimed to assess how some important characteristics and properties of conventional glass-ionomers were carried out according to the standards established by the ISO.

The selection of glass-ionomer is justified by the importance that the material gained since it was first proposed by Wilson and Kent more than 40 years ago^31^. With the advent of the Atraumatic Restorative Treatment (ART) ^32^, a procedure in which the cavity is cleaned only with hand instruments and restored with glass-ionomer cement, the restorative version of GIC became popular and a variety of GIC brands became available. Currently, the properties of glass-ionomer cements have become even more relevant because this material has been considered as a possible alternative to dental amalgams. Thus, although the mechanical properties of a product do not necessarily entirely indicate its clinical performance^28^, it is of paramount importance that a GIC has minimum standards set by recognized regulatory agencies before it can be considered appropriate for clinical use.

The results of this review show that, depending on the property studied, none or only one laboratory study universally followed valid standardized protocols proposed to test different GIC properties. Some authors stated that they followed the specifications, but they modified the dimensions of the test specimens, the storage times, and the speed of load application, therefore making it impossible to make direct comparisons with other studies^21^. For the compressive strength test, the test machine can be regulated at a cross-head speed of 0.75 mm/min and, provided the established parameters have been followed, the observed values can be compared. The test machine should always be calibrated to ensure reliable results. In this study, the speed of 0.75 mm/min was fixed and the interval of ±0.30 was not considered, since, if they were, any publication that had used speed between 0.45 and 1.05 mm/min would have been included. However, the data from these publications would not be comparable, due to lack of standardization, similar to studies with test specimens of different sizes. Therefore, the ISO should be asked to modify this important specification detail. Another important aspect that should be mentioned refers to how *in vitro* studies are reported. It was observed that the only study included in this review did not inform on whether sample randomization was performed or whether the evaluators were blinded to the procedures that each sample was submitted to, since it is not a requirement of the ISO specifications. However, as in randomized clinical trials, the randomization process in an *in vitro* experiment reduces the chances of bias and guarantees that the difference in outcome between groups is by chance. In addition, the inclusion of independent observers promotes transparency of the results^22^. This is quite relevant, and a checklist for reporting studies has already been proposed to improve the quality and transparency in reporting *in vitro* studies in experimental dental research^22^.

Unexpectedly, no studies regarding flexural strength, opacity, color and radiopacity survived the systematic review process and only one article for compressive strength could be included. This is of great concern as these properties are extremely important, since they are references for the indication of GICs for different clinical situations. Moreover, organizations such as the ISO seek to establish parameters to be observed before the introduction of new materials on the market. The Compressive Strength Test in particular is advocated by the ISO because most mastication forces are compressive in nature. The Compressive Fracture Strength (CFS) test is the only mechanical test established in ISO 9917 Part 1: Powder/liquid acid-base cements for hand-mixed GICs or conventional cements^24^. For resin-modified GICs, the ISO 9917 Part 2: Resin-modified cements^25^ do not recommend the CFS test, but the Flexural Strength (FS) test. Both tests can be considered satisfactory to evaluate the mechanical properties of restorative glass-ionomer cements and would represent stress-bearing in the clinical situation^9^. However, there is disagreement in the literature, considering that FS should be used rather than the CFS test, as GICs are brittle materials^23^. According to Baig and Fleming^21^ (2015), the only mechanical test that represents a discriminatory performance indicator for hand-mixed GICs is the CFS test, compared to the Three-Point Flexure Strength, Biaxial Flexure Strength and Hertzian Indentation tests. As mechanical properties are not intrinsic properties of GICs, details are very important and can produce completely different results for the same material if the guidelines are different. For example, the method of material preparation, discrepancies in the powder:liquid ratio, size of the specimens, storage time and duration, the loading rate used to perform the test etc., all affect the results obtained. Currently, the ISO specifications established for compressive strength and flexural strength specify a crosshead speed range of 0.75±0.30 mm/min and 0.75±0.25 mm/min, respectively, which means that the range for CFS is of the order of 0.45-1.05 mm/min and for FS it is 0.5-1.0 mm/min. This allows investigators flexibility from the lowest to the highest speed and the impact of this is that their results cannot be compared. It is suggested that the ISO should replace range values with point values in the tests and that researchers should strictly follow the specifications, so that the results, even though in different laboratories, can be compared.

The only article included in this review was the study conducted by Nomoto and McCabe^27^ (2001). They evaluated the effect of mixing methods on the compressive strength and porosity of GICs. Among the materials tested was a conventional restorative GIC presented in two forms: Ketac Molar Hand-mix and Ketac Molar Aplicap (ESPE, Seefeld, Germany). Both had P/L ratio below 3.5 and the results exceeded the minimum value of compressive strength stipulated by the ISO. The authors concluded that manual manipulation and the P/L ratios seem not to be the main factors interfering with the results. Other factors, including the differences in composition, viscosity and the incorporation of porosity must be considered. They concluded that the compressive strength test was able to distinguish changes in the mechanical properties of GICs through changes in composition and extent of porosity. The authors also observed that the FS test was initially an alternative to CFS but was rejected due to the difficulties in obtaining reliable specimens for testing.

In this review, a systematic search of literature for two other mechanical properties considered important to be evaluated for GICs was carried out: Diametral Tensile Strength (DTS) and Microhardness. However, due to the lack of international regulation and standardization to evaluate such properties, they were eventually excluded from this review. By carefully analyzing the articles that evaluated DTS, we found that most of them refer to the American Dental Association specification number 27,^33^ although this specification contraindicates this test. The ADA 27 specification relates to the ownership of “Tensile Strength (TS)”, which is very different from the property of “Diametral Tensile Strength (DTS)”.

The DTS test or “Tensile Strength by Diametral Compression” or “Brazilian Test” is the test adopted by the Brazilian Association of Technical Norms (ABNT) on the ABNT NBR 7222: 2011 registration^34^ for cylindrical specimens of concrete and not for dental cements. Therefore, there is an urgent need to: (1) come to a consensus about the relevance of testing GIC regarding DTS; and (2) to establish guidelines that can be applied globally with respect to the best way to test DTS.

According to the ISO 9917 Part 1^24^, to evaluate the optical properties for polyalkenoate restorative cements, the specimens should be prepared using a mold 1 mm thick and with 10 mm of internal diameter. Before measurements, the specimens must be stored for 7 days at 37°C in water in accordance with ISO 3696:1987^35^. The different studies on optical properties (color and opacity) in literature were not rigorously conducted using the ISO standard, and therefore were not included in this review. Twelve studies^36^ ^-^ ^47^ were excluded because the authors did not wait for the seven days required for specimens to be stored before analysis and four^48^ ^-^ ^51^ studies did the color analysis in specimens of 2 mm thick, making it impossible to compare results.

Regarding radiopacity, six articles were pre-selected, but five^52^ ^-^ ^56^ were excluded because the dimensions of the specimens did not follow ISO recommendations. One of them did not use the standard aluminum bar during radiographic shots and instead made specimens of different thicknesses^57^.

In summary, it was not possible to compare the results of mechanical and optical properties of conventional restorative GICs, because of a lack of standardization of the studies. It is very important that researchers adhere to the ISO specifications when planning and implementing laboratory experiments to allow comparisons to be made between different conventional restorative GICs.

## Conclusion

The scientific evidence that emerged from this review on the conventional restorative GIC test methodologies for mechanical and optical properties, which were compared in different GICs indicates that:

Only one published article that tested GIC mechanical and optical properties followed the ISO standards;

The study included was considered of fair quality, as the authors did not specify whether evaluators were blinded to the type of procedure/material that the sample was subjected to, and how samples were allocated to the different groups studied;

There is a need for ISO to replace the loading rate range with a loading rate standard for the mechanical tests for conventional restorative glass-ionomer cements;

There is a need for authors to strictly follow ISO instructions in laboratory experiments so that the data observed in different laboratories are comparable.
